# Comparison of *Schistosoma mansoni* Prevalence and Intensity of Infection, as Determined by the Circulating Cathodic Antigen Urine Assay or by the Kato-Katz Fecal Assay: A Systematic Review

**DOI:** 10.4269/ajtmh.15-0725

**Published:** 2016-03-02

**Authors:** Nupur Kittur, Jennifer D. Castleman, Carl H. Campbell, Charles H. King, Daniel G. Colley

**Affiliations:** Center for Tropical and Emerging Global Diseases, University of Georgia, Athens, Georgia; Department of Microbiology, University of Georgia, Athens, Georgia; Center for Global Health and Diseases, Case Western Reserve University School of Medicine, Cleveland, Ohio

## Abstract

The relationship between results from Kato-Katz (KK) fecal microscopy and urine-based point-of-care circulating cathodic antigen (POC-CCA) assays for *Schistosoma mansoni* infection remains a critical issue. This systematic literature review of 25 published papers compares prevalence of *S. mansoni* infection by KK with that by the POC-CCA assay. Nineteen published studies met our inclusion criteria for data extraction and analysis. Above a prevalence of 50% by KK, KK and POC-CCA results yielded essentially the same prevalence. Below 50% prevalence by KK, the prevalence by the POC-CCA assay was between 1.5- and 6-fold higher and increased as prevalence by KK decreased. Five of nine publications met inclusion criteria for extractable data on intensity of *S. mansoni* infection by KK assay and visual band density using the POC-CCA assay. A clear positive relationship exists between intensity by the KK and POC-CCA assays. This systematic review indicates that below 50% prevalence, the POC-CCA assay is much more sensitive than the KK assay. However, the existing data are inadequate to precisely define the relationship between POC-CCA and KK at lower levels of KK prevalence. More studies directly comparing the two assays in low-prevalence areas are essential to inform decision-making by national schistosomiasis control programs.

## Introduction

Planning for mass drug administration (MDA) to control *Schistosoma mansoni* requires accurate testing and mapping to determine local parasite prevalence. The present-day mapping approach is based on standardized stool microscopy to detect parasite eggs by the Kato-Katz (KK) smear technique.^1^,^2^ World Health Organization (WHO) guidelines for achieving control of infection-associated morbidity^1^ recommend different strategies and frequency of MDA with praziquantel, depending on the initial level of *S. mansoni* prevalence among school-aged children as determined by KK-based mapping. More recently, WHO guidance has focused on the possibility of achieving local elimination of *Schistosoma* infections in some areas. Because the KK assay is known to be less sensitive if prevalence and associated intensities of infection are low (or are being lowered due to multiple rounds of MDA),^3^–^5^ the KK assay may no longer be a sufficiently sensitive mapping tool in many places, particularly as the objectives of national programs move from morbidity control^6^ toward interruption of transmission.^7^,^8^

A commercially available, urine-based, point-of-care (POC), lateral flow cassette assay for the detection of a circulating cathodic antigen (CCA) of adult schistosomes has been on the market since 2008 and is now widely available. A number of published studies indicate that the POC-CCA assay may be much more sensitive in detecting *S. mansoni* infection than the KK stool assay, especially in areas of low prevalence.^9^–^16^ Latent class analysis of diagnostic characteristics in head-to-head trials has suggested that the POC-CCA assay has a considerably greater sensitivity and essentially comparable specificity as the KK assay.^13^,^14^

The current WHO guidelines^1^ are based on prevalence determined by KK assays. Because the relationship between prevalence based on POC-CCA and KK has not been clearly determined, these guidelines cannot be directly applied when POC-CCA is used for mapping, especially in areas of low prevalence, where the POC-CCA estimates are expected to be much greater than those based on KK. Also, programs often use measures of intensity based on KK egg counts to evaluate the effectiveness of control measures. Thus, as POC-CCA use increases, it will be important to determine whether the visual POC-CCA band intensities observed can also generally be used in this manner.

This systematic review of published data has two objectives. The first is to evaluate the relationship between prevalence determined by the KK assay and that obtained using the POC-CCA assay. The second is to compare the intensity of infection among infected individuals, as determined by the KK assay (in eggs per gram [EPG] of feces), to the density of the band in the POC-CCA assay as determined by visual evaluation.

## Materials and Methods

This systematic review was performed according to a search and analysis protocol we developed in March 2015 in response to queries from the Monitoring and Evaluation Group of the WHO Strategic and Technical Advisory Group for Neglected Tropical Diseases. A copy of the protocol is available from the corresponding author, Daniel G. Colley.

### Literature searches.

We conducted systematic electronic searches of PubMed and Web of Science through June 2015 according to recommended guidelines for meta-analysis.^17^ Our inclusion criteria were English language publications that evaluated *S. mansoni* prevalence by both the KK and the POC-CCA assays. No terms or key words were excluded, nor did we set parameters for publication dates, location, or study design. We used PubMed in our initial search, employing combinations of key search words, including *Schistosoma mansoni*, Kato Katz, POC-CCA, and diagnostics. In a typical search, we searched “All Fields” using the following search combination: schisto*and diag* and Kato-Katz or Kato Katz or KK and CCA or cathodic circulating antigen or POC-CCA. This search yielded 30 papers. We then searched Web of Science using a similar search combination, searching “TOPIC” with the search combination schisto* and diag* and Kato-Katz or Kato Katz or KK and CCA or cathodic circulating antigen or POC-CCA. This search yielded 66 papers. All 30 papers found in the PubMed search were also found in the Web of Science search. In addition, we hand searched the bibliographies of all collected papers. Three additional papers were discovered by this means.

We then applied the following exclusion criteria: reviews without prevalence data, republication of previously published data, papers that did not include *S. mansoni*, papers that did not assess prevalence of *S. mansoni* using both KK and POC-CCA, and papers that did not use the commercially available cassette for POC-CCA test (Rapid Medical Diagnostics, Pretoria, South Africa).

### Data abstraction.

Full texts for the 25 eligible papers were reviewed by two evaluators (Nupur Kittur, Jennifer D. Castleman). Regular meetings were held to discuss findings and discrepancies between the evaluators' assessments. A third author (Daniel G. Colley) resolved any differences in opinion.

Data on the following topics were abstracted and entered into a spreadsheet: study location, study population, treatment history, prevalence by KK, prevalence by POC-CCA, whether trace reading by POC-CCA was considered to be positive or negative, and whether the relationship between EPG and POC-CCA band intensity had been evaluated. Six papers were excluded at this stage based on lack of relevant comparisons of prevalence between KK and POC-CCA, because trace readings had been considered negative (contrary to the manufacturer's instructions) in calculating prevalence or because the study used the pre-commercial POC-CCA assay.

### Data synthesis.

Some of the included studies reported KK prevalence based on testing of a single stool sample per individual (two slides per stool), the approach typically used in mapping and control programs. Others provided results from up to three stools collected on different days, as is commonly done in research. One study only presented prevalence from two stool specimens and one only presented prevalence based on three stool specimens.

The POC-CCA is typically performed on one urine specimen per person. However, in one publication, prevalence was based on two urines collected on different days, and in another it was based on three urines collected on different days.

Data pairs representing *S. mansoni* prevalence by KK and the corresponding prevalence by POC-CCA were tabulated from the publications included in the systematic review. Several publications had more than one data pair because they reported prevalence separately for different epidemiological settings, different countries, or different age groups. The data pairs were divided into two groups: one with KK prevalence derived from a single stool sample (or the first stool sample if more than one was collected) and another with KK prevalence derived from more than one stool sample (usually three). GraphPad Prism 6 software (GraphPad Software Inc., La Jolla, CA) was used to perform linear regression analysis between prevalence by KK and prevalence by POC-CCA and to create box plots of the POC-CCA:KK prevalence ratio binned by KK prevalence. This analysis was done separately for the two groups of data pairs.

Five publications used graphs to present POC-CCA band intensities (0, trace, 1+, 2+, and 3+) versus EPG values in school-aged children. For these five publications, we estimated the median EPG obtained for each of these POC-CCA band intensities. The association between these KK and POC-CCA values was assessed visually by scatterplot using GraphPad Prism 6 software.

## Results

Figure 1

**Figure 1.**
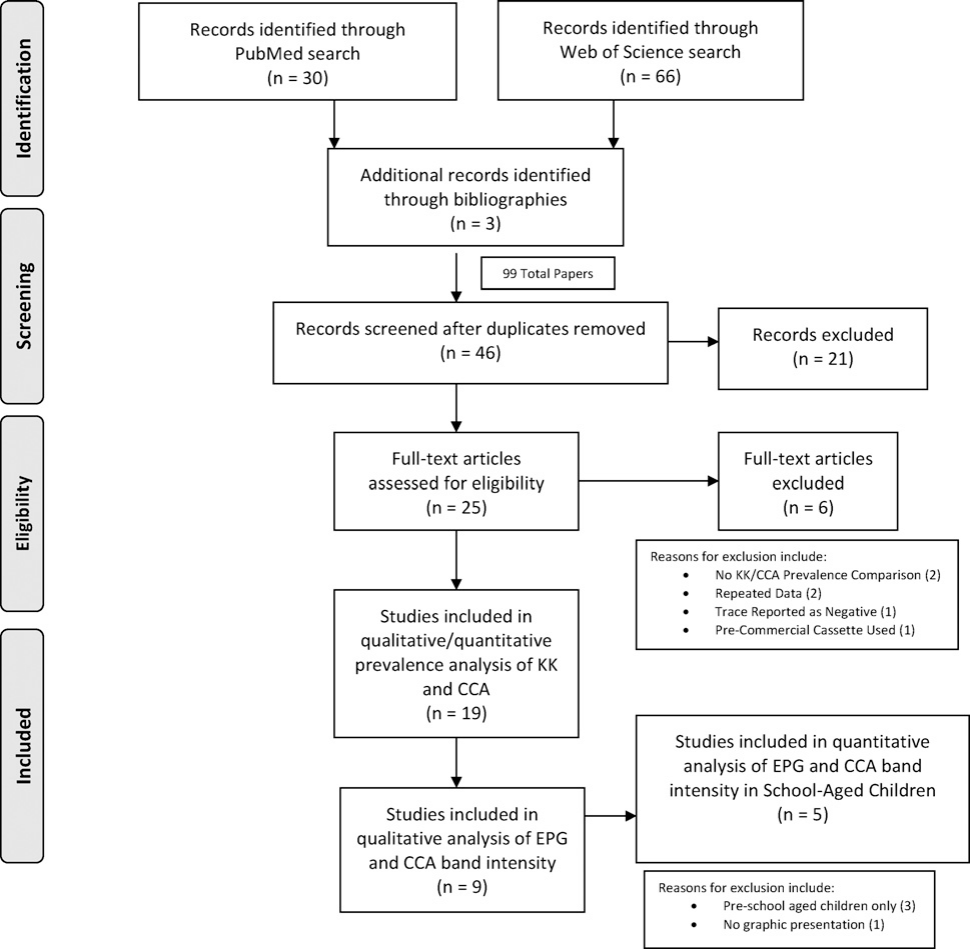
The PRISMA flow diagram for study selection.

presents the flow diagram of the literature search and evaluation.^18^ A total of 99 publications were identified on initial screening. Of these publications, 46 remained after removing duplicates and 21 were excluded based on our preestablished criteria. Twenty-five full-text articles were assessed for eligibility and six were excluded with reasons as indicated, leaving 19 for qualitative synthesis (see Figure 1). Details of these 19 publications are listed in Supplemental Table 1, which presents available data on study location, time of data collection, study population (ages and sample size), sample collection and method (number of specimens and slides where applicable), and whether the relationship between EPG and POC-CCA band intensity was evaluated.

**Figure 2.**
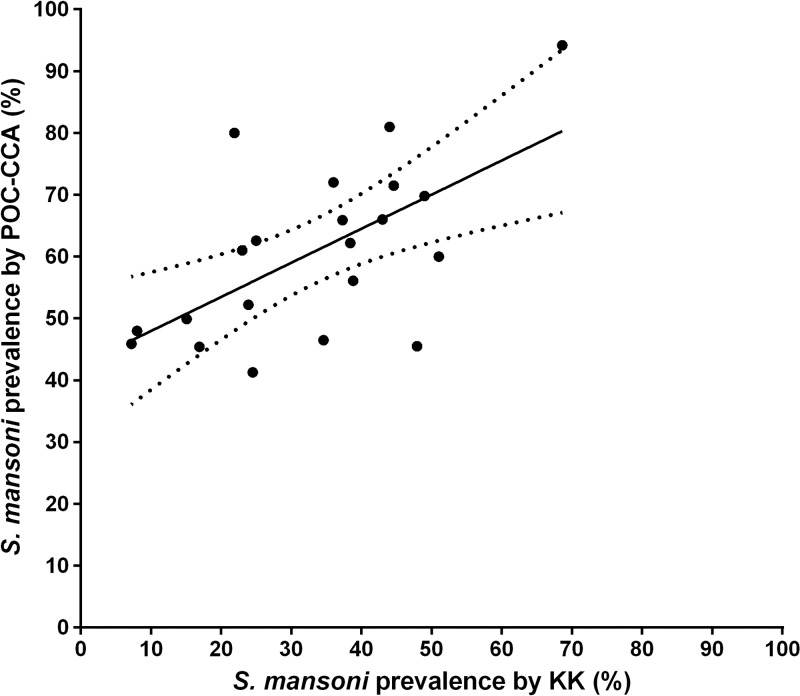
Scatterplot of *Schistosoma mansoni* prevalence as determined by a single point-of-care circulating cathodic antigen (CCA) urine assay vs. a single day Kato-Katz (KK) stool assay on the same participants. The solid linear regression trend line indicates the association between test values; the dotted lines indicate the 95% confidence interval for the observed regression: prevalence by POC-CCA = (prevalence by KK × 0.552) + 42.4%; *P* = 0.003.

Eleven studies provided 21 direct comparisons between one KK assay (single stool/two slides) and the POC-CCA assay. A scatterplot illustrating the relationship between *S. mansoni* prevalence based on these 21 KK versus POC-CCA comparisons is presented in Figure 2

. Figure 3

**Figure 3.**
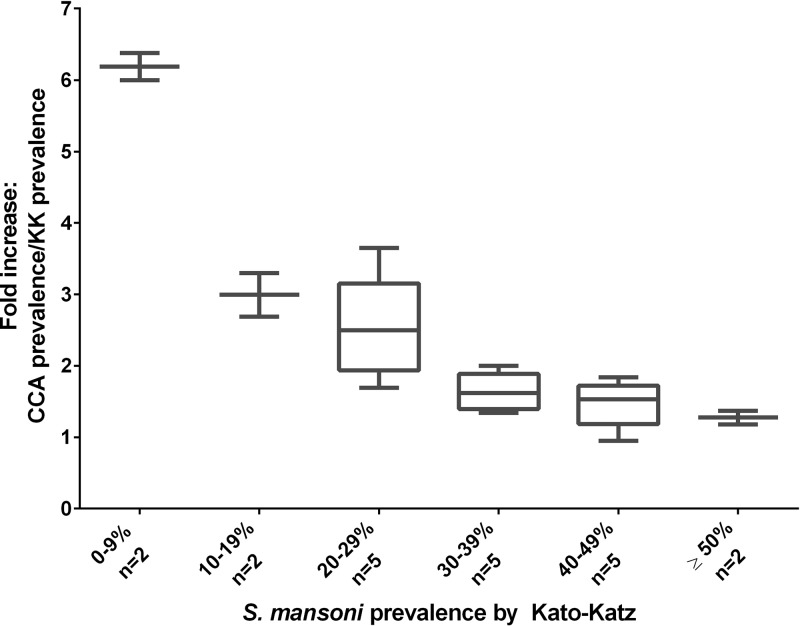
Box plots indicating the median, quartiles, and range of the effective multiplicative increases observed in prevalence when comparing single urine point-of-care circulating cathodic antigen (CCA) and single-stool Kato-Katz (KK) prevalence values for the same study populations (*y* axis), when classified according to ascending categories of observed KK prevalence (*x* axis).

presents these same data as box plots. This figure illustrates the nonlinear relationship between KK and POC-CCA estimates of prevalence. At 50% KK prevalence or above, prevalence by POC-CCA was nearly equivalent. However, when KK prevalence was below 50%, the prevalence by POC-CCA was consistently higher. Between 20% and 29% KK prevalence, the POC-CCA prevalence was, on average, 2.5-fold higher, and below 10% KK prevalence, the prevalence by POC-CCA was 6-fold higher.

**Figure 4.**
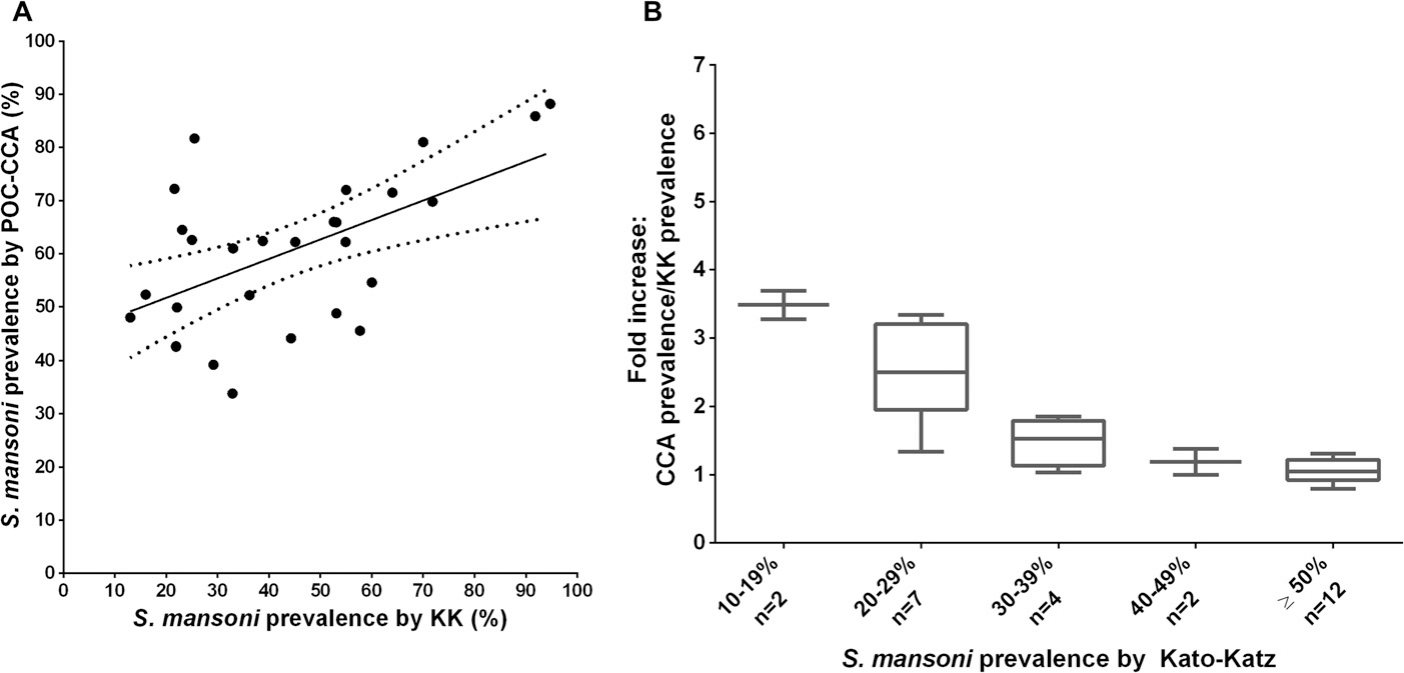
(**A**) Scatterplot of population prevalence as determined by a single point-of-care circulating cathodic antigen (CCA) urine assay vs. Kato-Katz (KK) stool assays for three separate days for the same subjects. The linear regression trend line indicates the association between test values; the dotted lines indicate the 95% confidence interval for the observed regression: prevalence by POC-CCA = (Prevalence by KK × 0.366) + 44.4%; *P* = 0.0025. (**B**) Box plots indicating the effective multiplicative increases observed in prevalence when comparing single urine POC-CCA and treble daily stool KK prevalence values for the same study populations.

Using 27 comparisons in the data from the 13 studies that included three daily stools (as is often done in research programs) yields essentially the same KK to POC-CCA relationship as comparing a single KK to a single POC-CCA (Figure 4A and B

). However, none of these involved locations with KK prevalence below 10%.

Figure 5

**Figure 5.**
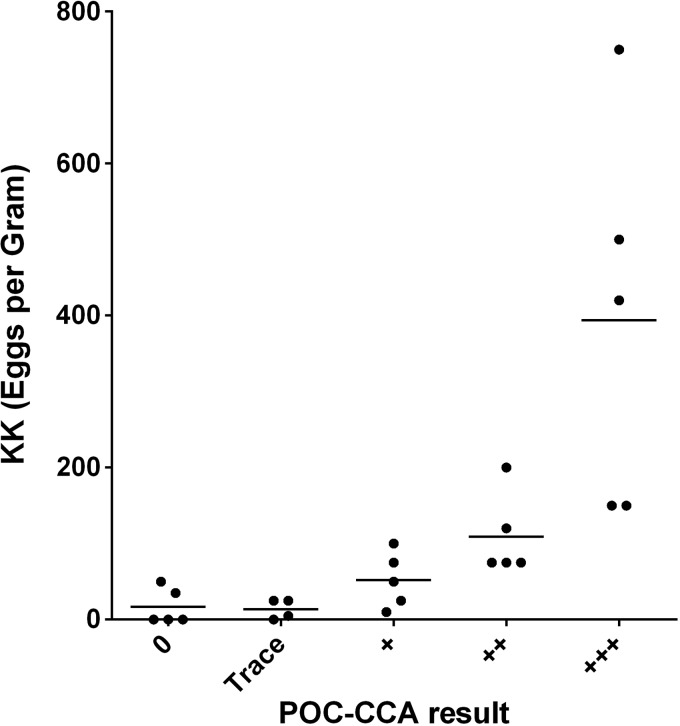
Scatterplot and median infection intensities (average eggs per gram feces, as determined by Kato-Katz assay) for subpopulations sorted according to observed antigen band densities detected on the point-of-care circulating cathodic antigen (CCA) urine assay (*N* = 5 publications). POC-CCA band densities were reported on a semiquantitative, ordinal scale as zero, trace, 1+, 2+, or 3+ values. Trace is considered a positive test according to the manufacturer's instructions.

presents the data abstracted from the five publications comparing visual density of the POC-CCA band result to the EPG in stool. There was an evident ordinal trend, with individuals with denser POC-CCA band readings having higher median EPGs than those with lower density POC-CCA bands.

## Discussion

Our systematic review demonstrates that there is a real relationship between KK and POC-CCA prevalence, but it is nonlinear. When the KK prevalence is above 50%, the POC-CCA and KK assays provide similar results. However, when the KK prevalence is lower, the estimate of prevalence by POC-CCA can be many-fold higher. In areas with POC-CCA prevalence below 30%, it appears that the KK prevalence could be less than 10% or even zero. Because only four of the studies were conducted in areas with less than 20% KK prevalence, further evaluation of test performance in low-prevalence areas is essential.

The findings related to the prevalence of *S. mansoni* obtained by parallel determinations using the KK stool assays and the POC-CCA urine assays were remarkably similar across 48 comparisons abstracted from 19 publications. Many of these studies were done by different investigators in different endemic areas, with different levels of prevalence and intensity of infection, and in different age groups with different treatment histories. Although the number of stools tested by KK varied from one to three, this did not appear to impact the association between POC-CCA and KK test results.

The POC-CCA uses urine instead of stool, results can be available immediately, and the cost of POC-CCA is either equivalent to or less than KK, depending on bulk pricing of the cassettes purchased.^19^ Therefore, schistosomiasis control programs are increasingly using it for mapping. Because the existing guidelines for programs are based on KK-based prevalence, programs need to be able to convert from POC-CCA to KK-based prevalence and vice versa. In addition, future guidelines are likely to incorporate both measures.

The current guidelines on schistosomiasis morbidity control^1^ have key cutoffs for deciding treatment regimens, which are 10% and 50% prevalence by KK. Our results suggest that in areas of high prevalence, the KK and POC-CCA assays both provide reasonable and comparable levels of prevalence and that the current guidelines are entirely adequate for morbidity control. However, because of the paucity of comparable, published data at levels of low KK prevalence, our findings are not robust enough to say with certainty what a given POC-CCA prevalence equates to in terms of KK at a KK prevalence below around 30%. Given that many areas are now at low-enough prevalence to aim for interruption of transmission, obtaining more comparison data in low-prevalence areas, especially those with KK prevalence of 0–20%, is urgent. It is hoped that ongoing and future studies, especially from low-prevalence areas will allow continued refinement of the direct relationships of prevalence levels obtained by these assays and other assays as they are developed and deployed.

A major limitation of our study is the relatively small number of studies that reported parallel *S. mansoni* prevalence by KK and the commercially available POC-CCA assay, which has only been on the market since September 2008. All the studies were conducted in Africa, which may have biased our findings. We could not determine the treatment history for participants in all the studies, which may affect prevalence. Only two of the studies included data on adults^20^,^21^; the rest were on preschool or school-aged children. In regard to our analysis of comparative intensities of KK and POC-CCA, we found seven publications, of which only five met our criteria; the other two concerned only preschool-aged children.^22^,^23^

Two additional caveats need to be considered in regard to the eventual utilization of the POC-CCA assay for mapping and monitoring of schistosomiasis control programs. One is the empirical finding that this assay is not reliable in detecting *Schistosoma haematobium*. This may be because *S. haematobium* worms do not produce as much CCA as *S. mansoni* worms, or *S. haematobium* CCA may be catabolized or cleared more efficiently. Thus, exclusive use of POC-CCA may miss areas with *S. haematobium*.

An additional issue about using the POC-CCA in *S. mansoni* control programs concerns the loss of the fecal microscopy for soil-transmitted helminths (STHs). National control programs using POC-CCA would still have to continue using KK or use other means to make decisions about STH interventions; however, the number of people who need to be tested for STH mapping is generally much less than that for schistosomiasis, so stool examinations might be done on a small subset of those tested using POC-CCA. In addition, sentinel sites could be designated for POC-CCA assay and KK testing for ongoing surveillance purposes.

Basing treatment strategies on the results of POC-CCA mapping in low-prevalence areas would, in the short term, lead to a recommendation to treat considerably more people. However, eliminating persistent low-level infections, one of the presumed sources of continued transmission, could speed up interruption of transmission. Nevertheless, research is also needed on the public health implications of high POC-CCA prevalence in areas with zero or near zero prevalence by KK.

The POC-CCA assay is proving a valuable tool in the effort to gain control, sustain control, and interrupt transmission of schistosomiasis. For it to be used optimally, more data are needed that clarify the relationship between results using this new method and other approaches. As new data become available, guidelines should be updated to support program managers to use the best tools for evidence-based decision making. Further research in low-prevalence areas is needed both to clarify the relationship between KK and POC-CCA results and to understand how best to manage places that have very low or no (0%) prevalence by KK, but significant prevalence using the POC-CCA or other new assays.

## Supplementary Material

Supplemental Table.
