# Supplementary material for: Comparison of Schistosoma mansoni Prevalence and Intensity of Infection, as Determined by the Circulating Cathodic Antigen Urine Assay or by the Kato-Katz Fecal Assay: A Systematic Review
Source: Am J Trop Med Hyg. 2016 Mar 2;94(3):605–10. doi: 10.4269/ajtmh.15-0725 (PMC4775897; doi:10.4269/ajtmh.15-0725)
Supplement: Supplementary file 1 [file SD9.pdf]

Supplemental Table 1

Included publications that compared Sm prevalence by KK and POC-CCA

Study	Study location (time of data collection)	Study population: age and sample size	Sample collection and methods	Relationship between EPG and CCA band intensity
Adriko and others[9]	Five primary schools in Bugiri District, shoreline of Lake Victoria, southeast Uganda	500 schoolchildren	Three stools, two KK slides each. Prevalence for one and two stools is also reported<br>One urine<br>Prevalence is reported for three settings: low-, moderate-, and high-endemic areas	N/A
Ashton and others 2011[24]	South Sudan (June–July 2010)	373 children aged 5–16 years	One stool, two KK slides each<br>One urine	N/A
Colley and others[14]	Cameroon, Cote d'Ivoire, Ethiopia, Kenya, Uganda (2010)	4,305 children aged 9–12 years	Three stools, two KK slides each. Prevalence for one stool is also reported<br>Overall prevalence is reported as well as separately for each country	N/A
Coulibaly and others 2013[25] (high-risk communities)	Two villages in Azaguie' District, south Cote d'Ivoire (June–September 2011)	209 + 158 preschool-aged children (< 6 years)	Two stools, two slides each<br>Two urines	N/A
Coulibaly and others 2013[27] (before and after treatment)	Two villages in Azaguie' District, south Cote d'Ivoire (August–November 2011)	242 preschool-aged children (< 6 years)	Two stools, two slides each	Data show a correlation between the color intensity of CCA (trace negative) test bands and EPG values
			Two urines with a scoring scheme to determine final CCA result	Excluded from graph because it focuses on preschoolers, and CCA reported in intensity comparison is trace negative result
Coulibaly and others 2012[26] (efficacy and safety of PZQ)	Two villages in Azaguie' District, south Cote d'Ivoire (June–November 2011)	160 preschool-aged children (< 6 years)	At least one stool sample with duplicate slides<br>One urine	N/A
Coulibaly and others[11] (accuracy of urine CCA)	Azaguie' District, south Cote d'Ivoire (October/November 2010)	146 children aged 8–12 years	Three stools, three slides each<br>Three urines<br>Prevalence is reported for 3 settings, two endemic for Sm (low and moderate) and third co-endemic for Sm and Sh	N/A
Dawson and others[22]	Walukuba and Piida, Buliisa District, on the shoreline of Lake Albert, Uganda (May/June 2011)	82 preschool-aged children	Two stools, two slides each	The box plot shows a significant positive association between EPG and the band intensity of the POC-CCA test
			One urine	Excluded from graph because it focuses on preschoolers
Erko and others 2013[28]	Jiga and Harbu towns, Ethiopia (2010–2011)	620 children aged 8–12 years	Three stools, Two slides each. Prevalence is reported for single, double, and six KK	CCA band color was intense in all children who had moderate and heavy intensity of infection. The odds ratio of strong intensity of the urine-CCA cassette test band color with an increase in EPG was statistically significant
			Three urines. Prevalence is reported for single and triple CCA	Excluded from the graph because no visual graphic was reported and no estimates could be extracted

(continued)

Supplemental Table 1
Continued

Study	Study location (time of data collection)	Study population: age and sample size	Sample collection and methods	Relationship between EPG and CCA band intensity
Lamberton and others 2014[29]	Mayuge District, Uganda (2004–2006)	76 children aged 6–12 years	Three stools, Two slides each	Trace is considered 1+. Used median estimates from baseline box plot in graph. Strong positive correlations were seen between the CCA band strengths and KK infection intensity categories
			One urine	Included in graph
Lodh and others[20]	Western Zambia	100 participants aged 18–50 years	One stool, two slides	N/A
			One urine	
Mwinzi and others[14]	Western Kenya (January 2013 to April 2014)	73 schoolchildren aged > 6 years	Stool samples were collected over 3 days (one stool, two slides per day) and prevalence is reported for each day and for all 3 days combined	There was positive association between intensity of infection by KK (EPG) and band intensity by POC-CCA: Spearman's $\rho = 0.601$ ($P < 0.001$)
			Urine samples were collected over 5 days and prevalence is reported for each day and for all 5 days combined	Included in graph
Shane and others[15]	Usoma, western Kenya (September–December 2007)	423 children aged 1–15 years	Three stools, two slides each	The average EPG found in each of the individuals was compared with band strength to determine if the band intensity of the CCA assays correlated with intensity of infection, and intensity was positively associated with intensity of infection ($P = 0.0001$)
			One urine	Included in graph
Sousa-Figueiredo and others[16]	Six lakeshore villages in Uganda (October/November 2009)	333 preschool-aged children (< 6 years)	One stool, two slides	N/A
			One urine<br>Prevalence is reported separately for low-, moderate-, and high-transmission settings	
Sousa-Figueiredo and others[21]	July 2007 in the Lake Albert region of Uganda<br>January/February 2009 in the Lake Victoria region of Uganda	125 preschool children and their mothers	Two stools, two slides each	N/A
			One urine<br>Prevalence is reported separately for mothers and children from two settings	
Standley and others 2010[30] (performance of CCA)	11 shoreline schools from eastern Lake Victoria in Tanzania and Kenya (January/February 2009)	171 children aged 6–17 years	One stool, two slides	For all the children surveyed, there was a very significant positive relationship between fecal EPG and CCA band intensity. Every additional 100 eggs led to a 6% increase in the likelihood of stepping up to the next intensity category of the CCA test band
			One urine	Included in graph
Standley and others 2010[31] (epidemiology and control)	Sesse Islands, Uganda (January 2010)	905 school-aged children	One stool, two slides	The box plot shows an overall positive increasing association between Sm EPG and the band intensity of the POC-CCA test with some outliers
			One urine	Included in graph

(continued)

Supplemental Table 1
Continued

Study	Study location (time of data collection)	Study population: age and sample size	Sample collection and methods	Relationship between EPG and CCA band intensity
Stothard and others[23]	Bugoigo on Lake Albert (April 2009)	242 children aged 5 months to 5 years	Two stools, two slides each	Data from this study show a strong correlation between the Sm EPG and intensity of CCA test bands
			Two urines, one CCA on most children	Excluded from the graph because it focuses on preschoolers
Tchuente and others 2012[32]	Cameroon (December 2010 to January 2011)	138 children aged 8–12 years	Three KK, three slides each. Prevalence is reported for one and three KK<br>Three urines. Prevalence is reported for one and three CCA	N/A

CCA = circulating cathodic antigen; EPG = eggs per gram; KK = Kato-Katz; N/A = not applicable; POC = point-of-care; PZQ = praziquantel; Sh = *Schistosoma haematobium*; Sm = *Schistosoma mansoni*.
